# Immune status & enzymes activity in blood lymphocytes in adult patients at different stages of acute lymphoblastic leukaemia

**Published:** 2011-03

**Authors:** Olga V. Smirnova, Valery T. Manchouk, Andrey A. Savchenko

**Affiliations:** *Institute of Medical Problems of the North, Siberian Division, Russian Academy of Medical Sciences, Krasnoyarsk city, Russia*

**Keywords:** Acute lymphoblast leukaemia, enzymes, immune status, lymphocytes

## Abstract

**Background & objectives:**

Pathogenesis acute lymphoblastic leukaemia (ALL) in adults is not well understood, as it is more common in children. We examined the immunological status and the activity of certain enzymes in blood lymphocytes in adult patients of ALL at different stages.

**Methods:**

ALL patients (n=71) admitted during 2000-2005 were included in this study. All patients had decreased T-lymphocytes content. At first attack, they had CD4 ^+^-cells decreasing and increasing IgM and IgG concentration. In complete remission all examined parameters were low. The peculiarities of ALL recurrence were high NK-cells content and disbalances of the main immunoglobulin concentrations.

**Results:**

In the first attack and recurrence the anaerobe glucose oxidation intensity and the reactions of macromolecular synthesis were lower in lymphocytes compared to control. In remission all these processes restored to normal. In all stages in lymphocytes GR had decreased activity.

**Interpretation & Conclusions:**

Our results showed that most of changes in immune status of ALL patients were in a stage of complete remission when patients arrived on its maintenance through the small period from spent before therapy when the immune system of the patient has not been restored. Thus, probably cytostatic action causes immune failure in the future and starts disease again.

The frequency of acute lymphoblast leukaemia (ALL) in different countries has been reported to be 2-5 cases per 100 000 people a year^1^,^2^. Children often suffer from ALL, but among adults this form acute leukemia is seen in 10-15 per cent patients. Complete remission achived in 75-80 per cent adult patients, but only 30 per cent of them survive 5 years after the beginning the disease^1^–^3^. ALL in adults is severe and more malignant compared to children. Further, recurrence has poor prognosis than the previous one, and the appearance of recurrence decreases recovery^1^,^2^,^4^–^7^.

Many investigations have been done on the clinical and medical aspects of these patients^2^,^3^. But pathogenic aspects of this disease are not well studied, particularly the immune mechanisms causing the development of complications and their role in the progression of ALL. It is known that functional activity of lymphocytes is determined by their population and subpopulation content and by the state of metabolic intracellular processes, which are responsible for necessary aspects^8^–^10^. we carried out this investigation to study the immune status and activity of enzymes in blood lymphocytes in adult patients with different ALL stages.

## Material & Methods

All consecutive patients with common variant of ALL who were admitted in Hematology department of Regional Clinical Hospital №1 Krasnoyarsk city, Russia, during 2000 to 2005, were included in the investigation. Prior to the sample being taken, informed consent was obtained from all subjects. The study protocol was approved by the Ethics Committee of Institute of Medical Problems of the North and by the Ethics Committee of Regional clinical hospital №1 Krasnoyarsk city. A total of 71 ALL male patients in age range 35-65 yr were included; 23 patients were in first attack, 26 patients were in the stage of complete remission after treatment using programme of cytostatic therapy and 22 patients were in recurrence. Normal healthy adults (n=106) of the same age group constituted the control group.

On admission the anamnesis was gathered and the complete clinic-laboratory investigation was performed in all patients to role their clinical signs. The severity of disease is an expression of clinical symptoms display. All patients had sternum puncture for percentage and amount cells determination, cytochemical estimation of enzymes and immune sternal punctuate phenotype after admission.

The patients were divided into 3 groups: patients in a stage of primary attack, patients in a stage of complete remission, and patients in a stage of repeated recurrence. In patients who were in a stage of complete remission, the blast quantity in sternum puncture was 1-3 per cent, it varied from 25 to 96 per cent in patients in a stage of primary attack and from 26 to 89 per cent in patients in a stage of repeated recurrence. All patients had the sternum puncture of bone brain to assess the quantitative parameters of cells, calculations were made on 1000 cells, percentage was defined. The patient treatment was made adequately under programme of cytostatic therapy. All laboratory blood tests for estimation of the immune status and activity of lymphocytes enzymes were done before starting treatment. The 5 per cent and less blast cells in sternum bone brain with its normal cells content, absence of leukaemia cells in spinal liquid were criteria of complete remission for ALL patients in our research^1^,^2^. Recurrence was diagnosed if >25 per cent blast cells were determined in sternum bone brain in patients in remission. Blood (10-15 ml) was drawn from patients and lymphocytes were separated. The immune status in patients was determined by method of indirect immune fluorescence with use monoclonal antibodies to CD3, CD4, CD8, CD16, CD19, HLA-DR. Additionally we determined immune regulatory index (CD4^+^/CD8^+^) and index of T-lymphocytes activation (HLA-DR^+^/CD19^+^). Reagents were immunoglobulin A, M and G concentrations were calculated by immunoenzyme method. The state of humoral immunity also was estimated using indices of Ig A (Ig A/CD19^+^), Ig M (Ig M/CD19^+^), Ig G (Ig G/CD19^+^) synthesis. The test system Vector-Best from Russia was used^11^.

The definition of activity of NAD (P)-dependent dehydrogenases was made by using bioluminescent method by earlier developed techniques^6^. Using this method, activities of enzymes: glucose-6-phosphate dehydrogenase (G6PDG, malic enzyme (NADPMDG), NAD- and NADH-dependent lactate dehydrogenase (LDG and NADH-LDG), NAD- and NADH-dependent malate dehydrogenase (MDG and NADH-MDG), NADP- and NADPH-dependent glutamate dehydrogenase (NADP-GDG and NADPH-GDG), NAD- and NADH-dependent glutamate dehydrogenase (NAD-GDG and NADH-GDG), NAD- and NAD-dependent isocitrate dehydrogenase (NAD-ICDG and NADP-ICDG), and glutathione reductase (GR) were studied. The activity of dehydrogenases in blood lymphocytes was calculated in enzyme units (1U=1mkmol/min^3^ on 10^4^ cells.

All data were presented as mean ± SEM. The data were not normally distributed; Mann-Whitney non-parametric test and Kruskal – Wallis ANOVA were applied.

## Results and Discussion

At first attack of ALL the percentage and absolute content of lymphocytes in blood were higher, but percentage CD3^+^-cells amount was lower (Table I). The patients in first attack showed an increase in absolute CD8^+^- and CD19^+^-lymphocytes content, and a decrease of absolute CD16^+^-level and the percentage and absolute HLA-DR^+^-cells content. Also these patients had high index of T-lymphocytes activation and small immune regulatory index.

**Table I:** Cellular immune status in patients with different stages of ALL

Indicators	Control group	Patient group		
	N=106	Attack N=23	Remission N=26	Recurrence N=22

Leukocytes, (10^9^/l)	6.40 ± 0.16	6.08 ± 0.83	5.79 ± 0.42	6.06 ± 0.20
Lymphocytes, (%)	38.5 ± 0.8	47.6 ± 6.2**	27.8 ± 2.3***†††	42.2 ± 4.8δδ
Lymphocytes, (10^9^/l)	2.27 ± 0.06	2.84 ± 0.42*	1.61 ± 0.16***††	2.64 ± 0.23*δδδ
CD3^+^, (%)	66.6 ± 0.6	57.5 ± 3.6**	56.6 ± 1.9***	58.9 ± 2.9**
CD3^+^, (10^9^/l)	1.48 ± 0.05	1.53 ± 0.25	0.85 ± 0.07***†††	145 ± 0.19δδ
CD4^+^, (%)	41.5 ± 0.8	33.4 ± 3.5**	34.9 ± 1.0***	44.5 ± 2.3††δδ
CD4^+^, (10^9^/l)	0.93 ± 0.04	0.98 ± 0.18	0.48 ± 0.06***†	1.00 ± 0.07δδδ
CD8^+^, (%)	26.6 ± 0.7	27.2 ± 2.5	21.8 ± 1.4**	28.0 ± 2.1δδ
CD8^+^, (10^9^/l)	0.62 ± 0.02	1.19 ± 0.22***	0.25 ± 0.02***	0.73 ± 0.10δδδ
CD16^+^, (%)	19.5 ± 0.5	17.5 ± 2.0	17.7 ± 2.4	24.1 ± 2.2***†δ
CD16^+^, (10^9^/l)	0.47 ± 0.02	0.38 ± 0.07*	0.31 ± 0.06***	0.57 ± 0.07δδ
CD19^+^, (%)	12.5 ± 0.4	10.6 ± 1.8	10.9 ± 1.5	12.7 ± 1.9
CD19^+^, (10^9^/l)	0.28 ± 0.01	0.40 ± 0.09*	0.15 ± 0.02***†††	0.21 ± 0.03*
HLA-DR^+^, (%)	15.9 ± 0.5	9.8 ± 1.2***	16.9 ± 2.2†	25.8 ± 3.9*δ††
HLA-DR^+^, (10^9^/l)	0.37 ± 0.02	0.18 ± 0.02***	0.18 ± 0.02***	0.42 ± 0.07††δδ
HLA-DR^+^/CD19^+^	1.23 ± 0.05	1.60 ± 0.09**	1.47 ± 0.12*	2.15 ± 0.17***††δδ
CD4^+^/CD8^+^	1.51 ± 0.04	1.26 ± 0.08*	1.43 ± 0.07	1.73 ± 0.10†††δ

Values are mean ± SEM

P^*^<0.05

** <0.01

*** <0.001 compared to control;

*P*^†^<0.05

†† <0.01

††† <0.001 compared to attack value;

*P*^δ^<0.05

δδ <0.01

δδδ <0.001 compared to remission

In complete remission, the patients revealed a decrease in percentage and absolute content of lymphocytes, CD3^+^-, CD4^+^- and CD8^+^-cells. Also these patients had the low absolute CD16^+^-, CD19^+^- and HLA-DR^+^-lymphocytes content. In remission, index of T-lymphocytes activation was higher (Table I).

In recurrence many indicators of cellular compartment of immune system became normal. For example, the absolute CD3^+^-, CD16^+^- and HLA-DR^+^-cells content, the percentage and the absolute CD4^+^- and CD8^+^-lymphocytes content were higher than remission and became normal. Also like in first attack, in complete remission the patients had the low percentage CD3^+^-lymphocytes level. The absolute CD19^+^cells content in these patients remained low relative to the control values. The percentage CD16+- and HLA-DR+-lymphocytes amount, index activation of T-lymphocytes in patients in recurrence were higher relative the values in attack, remission and became normal.

The indicators of humoral immunity also depended on stages of ALL (Table II). In first attack Ig M and Ig G concentrations were higher, but indices of Ig A and Ig G synthesis were lower. In remission, low Ig G concentration was seen, there was increase in index of Ig A synthesis relative to the control and first attack values, and the increase of index of Ig G synthesis compared to the first attack values. In recurrence low Ig A and Ig M serum concentration and increase of serum Ig G content were seen. In this stage all indices of Ig A, Ig M and Ig G synthesis were lower (Table II).

**Table II:** Humoral immune status in patients with different stages of ALL

Indicators	Control group	Patient group		
	Control N=106	Attack N=23	Remission N=36	Recurrence N=22

Ig A, (g/l)	2.23 ± 0.08	1.88 ± 0.32	2.18 ± 0.39	0.92 ± 0.08***†δ
Ig M, (g/l)	1.20 ± 0.06	1.61 ± 0.23*	1.14 ± 0.16	0.58 ± 0.11***†††δδ
Ig G, (g/l)	10.94 ± 0.32	16.71 ± 2.81**	6.70 ± 0.50***††	18.54 ± 3.53**δδ
Ig A/CD19^+^, (ng/cell)	8.65 ± 0.71	3.63 ± 0.68**	14.73 ± 3.22**††	2.42 ± 0.40***δδδ
Ig M/CD19^+^, (ng/cell)	7.39 ± 0.78	4.50 ± 1.29	6.15 ± 1.04	0.88 ± 0.16***†δδδ
Ig G/CD19^+^, (ng/cell)	39.43 ± 2.33	18.36 ± 2.72***	42.10 ± 5.25†††	21.01 ± 1.30***δδδ

Values are mean ± SEM

* *P*<0.05

** <0.01

*** <0.001 compared to control;

† *P*<0.05

†† <0.01

††† <0.001 compared to attack value;

δ *P*<0.05

δδ <0.01

δδδ <0.001 compared to remission

At the first attack, there were decreased levels of T-lymphocytes, CD4^+^- and NK-cells content and immune regulatory index was low. At this stage there was decrease in HLA-DR^+^-cells. Using index of T-lymphocytes activation it was observed that these patients had the increasing active T-lymphocytes content.

The complete remission was characterized by severe disturbances of immune status in ALL patients. T-lymphocytes, regulatory fractions of T-lymphocytes, NK-cells and B-lymphocytes were lower in all patients. Immune regulatory index became normal as proportional of CD4+-cells and cytotoxic T-lymphocytes levels decreased. HLA-DR^+^-cells content in patients in remission was normal. Ig G concentration was low and B-cells function restored to normal.

In recurrence, T-lymphocytes levels were low but the content of regulatory T-lymphocytes fractions was normal. The peculiarity of this stage was the increasing NK-cells and HLA-DR^+^-lymphocytes content. Active T-lymphocytes level was high.

**Fig. 1:**
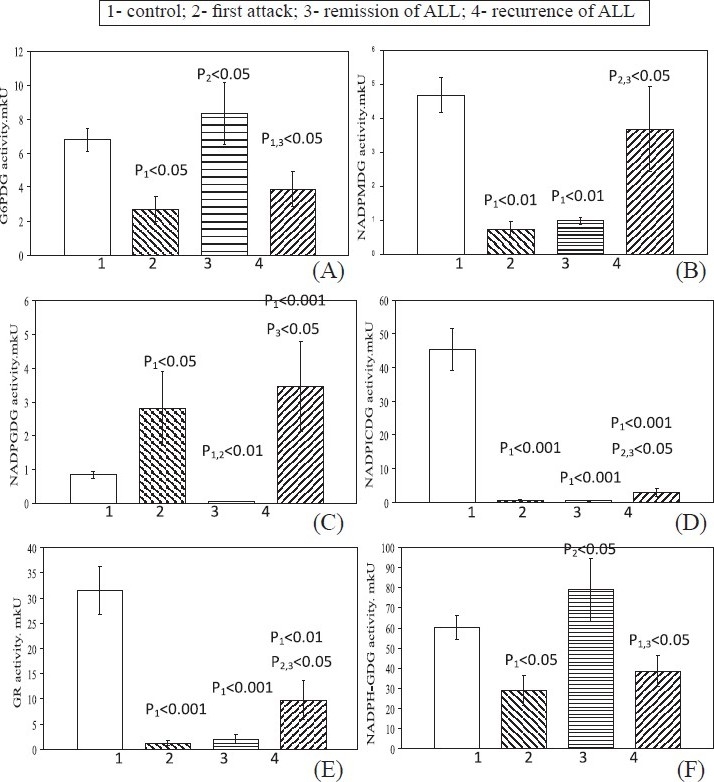
Levels of NADP-dependent dehydrogenase activities in blood lymphocytes in patients with different stages of ALL. Values are mean ± SEM.

During first attack in blood lymphocytes there were significant decrease in levels of G6PDG (Fig. 1 A), NADPMDG (Fig. 1 B), NADPICDG (Fig. 1 D), GR (Fig. 1 E) and NADPH-GDG (Fig. 1 F) and a significant increase in level of NADPGDG (Fig. 1 C). In complete remission, NADPMDG, NADPICDG, and GR activities were low. Same time the G6PDG and NADPH-GDG activity were higher and became normal in these patients. The NADPGDG activity in blood lymphocytes in patients in ALL remission was low relative to the first attack and control values.

In recurrence, like in first attack, the G6PDG and NADPH-GDG activities were lower. The NADPGDG activities was higher than control and remission (Fig. 1 A, F). The NADPMDG activity became norm. The levels of NADPICDG and GR activities were higher relative to the first attack and remission values, but remained low relative to the control values. (Fig. 1 D, E)

**Fig. 2:**
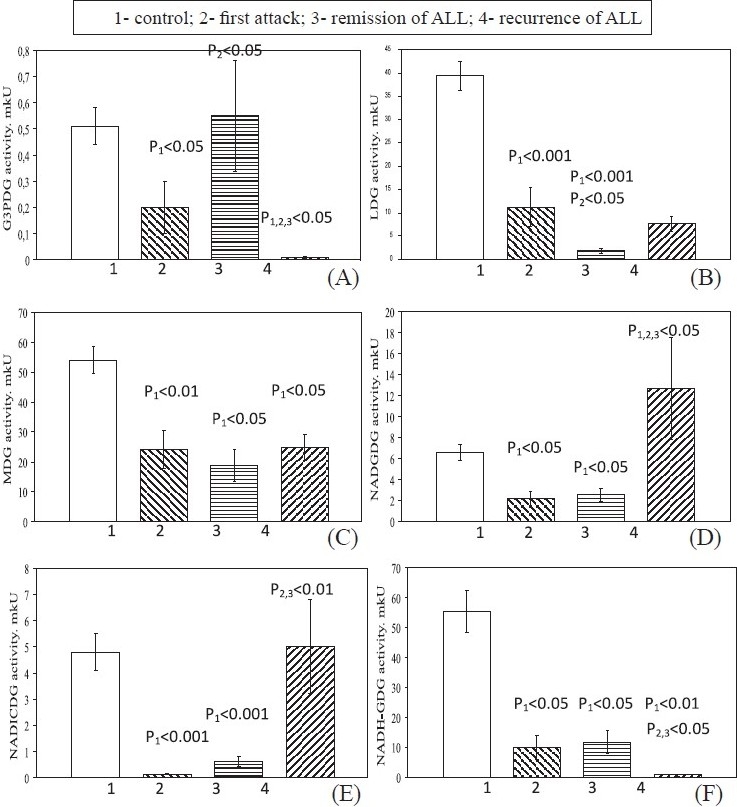
Levels of NAD-dependent dehydrogenase activities in blood lymphocytes in patients with different stages of ALL. Values are mean ± SEM.

In first attack G3PDG (Fig. 2 A), LDG (Fig. 2 B), MDG (Fig. 2 C), NADGDG (Fig. 2 D), NADICDG (Fig. 2 E) and NADH-GDG (Fig. 2 F) levels decreased compared to control. In remission, the MDG, NADGDG, NADICDG and NADH-GDG activities remained low relative to the control values. The LDG activity in blood lymphocytes became significantly low than in the first attack. G3PDG activity was restored to the control values. In recurrence, the MDG activity remained low relative to the control values. The G6PDG and NADH-GDG activities were significantly lower relative to the control, first attack and remission values. The activity of LDG in lymphocytes in recurrence was higher relative to the remission values and remained low relative to the first attack and control values. The activity of NADGDG in recurrence was higher relative to the all stages values. The NADICDG activity was a little higher to control indicators.

**Fig. 3:**
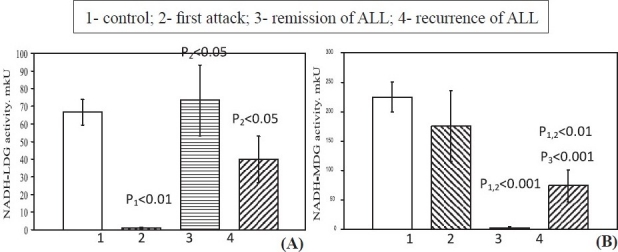
NADH-dependent reactions LDG (A) and MDG (B) activities in blood lymphocytes in patients of ALL. Values are mean ± SEM.

The NADH-LDG activity in blood lymphocytes in first attack was lower, in remission it was restored to the control values and remained the same in recurrence (Fig. 3 A). The NADH-MDG activity in first attack of ALL remained normal, but in remission it was significantly lower (Fig. 3 B) than in control and attack). In recurrence of ALL, the activity of this enzyme was higher relative to the remission values, but was remained statistically low relative to the control and first attack values.

These enzymes occupy key position in different immune competent cells metabolic pathways^1^,^6^. The decreased intracellular G6PDG activity in patients of ALL in first attack and recurrence led to little output of products of pentose phosphate cycle and inhibited many reactions of macromolecular synthesis. G6PDG is the main enzyme of glycolysis. We proposed that patients in first attack and recurrence had the anaerobic glycolysis inhibition in terminal reactions. The G3PDG activity, responsible for the transfer of the products of lipid catabolism to reactions of glycolysis, in patients in attack and in recurrence also, was lower. In remission the level of this enzyme was restored to normal, so in this stage we observed the normal reactions of anaerobic glucose oxidation.

The activity of GR in first attack and recurrence was lower because of low level of NADPH output in pentose phosphate cycle. In remission there was a significant decrease of NADH-GDG activity, on which the synthesis of glutathione depends. So there was increasing level of peroxidating processes in lymphocytes decreasing their functional activity.

Lymphocytes are aerobic cells. It is known, that intensity of aerobic processes depends on cycle of tricarbonic acids activity. The activity of NADICDG, which responded for early reactions, was significantly lower in blood lymphocytes in patients in first attack and complete remission. Same time, MDG activity was lower in all stages of ALL. The level of lacertus reaction of NADPICDG activity also was lower in all stages of disease.

In all ALL patients aerobic LDG reaction was lower. NADPMDG reaction was lower in first attack and complete remission and became normal in recurrence. There were connections from amino acid metabolism reactions to with the help of NADGDG and NADPGDG. In patients during first attack and complete remission NADGDG activity was lower, but in recurrence it became high. The NADPGDG reaction was higher in attack and recurrence

In blood lymphocytes of ALL patients in all stages the intensity of aerobic processes was lower, the processes of substrate connections between reactions of cycle of tricarbonic acids and amino acids metabolism reactions were disturbed. In remission and recurrence, the disturbances of metabolic status of mitochondria compartment was complicated by the inhibition of the key malat-aspartat shunt reaction and hydrogenous gradient was lower^1^–^3^. Having analyzed changing activity of lymphocyte enzymes in different ALL stages, and, also having connected them with features of a clinical picture of patients, we could predict the occurrence of infectious and haemorrhagic complications in these patients under the initial enzyme status of lymphocytes. (patent no. RU2315 305C2, RU 2324 190 C2).

Thus, during the examination of immune system and activities of enzymes in blood lymphocytes it was found that all patients with ALL had the decreasing T- lymphocytes content in blood. In first attack, the CD4^+^- lymphocytes content was lower with increased Ig M and Ig G concentration. In complete remission, all examined parameters were lower. The peculiarities of recurrence ALL was the high NK-cells content and disbalance of the main immunoglobulin concentrations. Our results showed that most of changes in immune status of patients were in a stage of complete remission when patients arrived on its maintenance through the small period from spent before therapy when the immune system of the patient has not been restored. Perhaps cytostatic action causes immune failure later on and starts disease again. Probably, use in maintenance of remission of replaceable therapy will allow adapting the patient for the subsequent pathogenetic treatment and will not cause repeated recurrence of disease
